# Monitoring of Influenza Vaccination Coverage among Pregnant Women in Germany Based on Nationwide Outpatient Claims Data: Findings for Seasons 2014/15 to 2019/20

**DOI:** 10.3390/vaccines9050485

**Published:** 2021-05-11

**Authors:** Annika Steffen, Thorsten Rieck, Anette Siedler

**Affiliations:** Immunization Unit, Robert Koch Institute, 13353 Berlin, Germany; rieckt@rki.de (T.R.); siedlera@rki.de (A.S.)

**Keywords:** claims data, Germany, influenza, pregnancy, vaccination coverage

## Abstract

Pregnant women and their infants are at increased risk for severe influenza-related complications. A decade has passed since influenza vaccination was first recommended for pregnant women in Germany in 2010; however, monitoring of vaccination coverage (VC) has not yet been implemented for this target group. Using nationwide outpatient claims data, we here provide results on influenza VC among pregnant women in Germany for seasons 2014/15 to 2019/20. For any given season, pregnant women were defined as women who had undergone prenatal health care in at least two consecutive quarters within a season. VC increased from 9.0% in season 2014/15 to 16.6% in 2019/20 (+84%), while most of the increase occurred from season 2016/17 (VC: 9.9%) onwards (+68%). Consistently across seasons, women in east Germany were 40 to 60% more likely to be vaccinated compared to women residing in west Germany. According to age, the highest VC was observed among women aged 35 to <40 years (2019/20: 18.2%). Despite noticeable increases in influenza VC during recent years, overall coverage remains low among pregnant women. Starting with this analysis, VC among pregnant women in Germany will be monitored on a yearly basis in order to detect trends and identify immunization gaps.

## 1. Introduction

Pregnant women and their infants are at increased risk for severe influenza-related complications and hospitalization [1]. The vaccination of pregnant women against seasonal influenza has therefore become a key strategy to prevent influenza and influenza-related complications in pregnant women and their infants in many high-income countries [2,3,4]. Compelling evidence indicates that influenza vaccination during pregnancy is safe and effective in preventing influenza illness and related complications in both pregnant women and their newborns [5,6,7]. Despite this, vaccination coverage (VC) has generally been observed to be suboptimal [2,8,9,10]. In the European Union (EU) and European Economic Area (EEA), for instance, the median VC was 25% in season 2016/17, ranging from 0.5% in Slovenia to 59% in Northern Ireland [2,10]. Of note, although 28 of 30 EU/EEA Member States recommend routine immunization of pregnant women against seasonal influenza, a monitoring system of VC has rarely been implemented and estimates for the EU/EEA region are solely based on nine Member States [2,10].

In Germany, the National Immunization Technical Advisory Group (Standing Committee on Vaccination, STIKO) recommends vaccination against seasonal influenza for individuals aged ≥60 years, individuals with underlying chronic diseases, residents of nursing homes, healthcare workers, and, since 2010, pregnant women [11]. Specifically, all pregnant women should be vaccinated against seasonal influenza from the second trimester, and in case of an underlying chronic condition, from the first trimester onwards [12]. The annual influenza vaccination is free of charge for all population subgroups included in the STIKO recommendation and fully covered by statutory health insurance (SHI), which comprises about 87% of the population in Germany. In 2004, a nationwide immunization information system for the monitoring of VC based on outpatient claims data comprising all SHI-insured individuals was launched [13]. From 2019 onwards, VC of all vaccinations as recommended by the STIKO has been published on an annual basis [14,15]. Although a decade has passed since influenza vaccination was first recommended for pregnant women in Germany, a regular monitoring of VC in this target group has not been implemented yet. So far, data on national influenza VC during pregnancy is limited to a few studies covering single [16,17,18] or a few consecutive seasons [19].

In an effort to address the lack of VC monitoring among pregnant women in Germany, the present study is the prelude to annual reporting of VC in this target group. Using nationwide outpatient claims data, we here provide recent data on influenza VC among pregnant women for seasons 2014/15 to 2019/20. By this, we aim (a) to evaluate how well recommendations have been implemented in practice and (b) to monitor trends in VC over time. The knowledge derived from these data may further guide the development and refinement of tailored strategies to increase vaccination uptake and, ultimately, to reduce the burden from influenza-related complications during pregnancy.

## 2. Materials and Methods

### 2.1. Data Basis

For the present analysis, we used nationwide outpatient claims data of all statutory health insured (SHI) individuals, covering 87% of the German population. The data include date-specific information on outpatient vaccinations and diagnoses by quarter, outpatient services according to the Doctors’ Fee Scale within the SHI scheme, as well as information on the patient’s year and month of birth, sex, and region of residence (17 regions representing the different Associations of Statutory Health Insurance Physicians, ASHIPs, in Germany). With the exception of North-Rhine Westphalia, which consists of two ASHIP regions, the administrative ASHIP regions correspond to the 16 federal states. As the availability of necessary data differed between ASHIPs, we included data from 16 ASHIPs in the analyses of seasons 2017/18 to 2019/20, while data from 13, 14, and 15 ASHIPs were included in the analyses of seasons 2014/15, 2015/16 and 2016/17, respectively. Hence, our analysis was based on data covering 66% (2014/15) to 80% (from 2017/18 onwards) of the total population in Germany, respectively.

### 2.2. Definition of Pregnancy during Influenza Season

In Germany, prenatal care including pregnancy-related vaccination is guided by maternity care guidelines that define a nationwide standardized program for all pregnant women. This program is fully funded by health insurance and primarily conducted by office-based gynecologists [20]. Pregnancy-related health care services covered by the program are coded according to the Doctors’ Fee Scale using the position “01770”. This code can be used for the purpose of billing services of prenatal care once per calendar quarter of pregnancy.

Hence, we defined pregnancy on a quarterly basis based on outpatient services for prenatal care coded as “01770” among women of childbearing age (15 to 49 years). For any given influenza season (third quarter until first quarter of the following year), women who were pregnant during the season and thus eligible for influenza vaccination were defined as women who had undergone prenatal examinations in at least two consecutive quarters of that season.

### 2.3. Calculation of Vaccination Coverage

For each season, VC was defined as the number of women who were pregnant during the respective season and received influenza vaccination during that season over the total number of women who were pregnant during that season. VC was calculated for Germany as a whole, as well as according to region (east vs. west) and age group (15 to <20, 20 to <25, 25 to <30, 30 to <35, 35 to <40, and 40+ years). For the comparison of east and west Germany, ASHIPs were classified into eastern (Berlin, Brandenburg, Mecklenburg-Western Pomerania, Saxony, Saxony-Anhalt and Thuringia) and western federal states (Baden-Wuerttemberg, Bavaria, Bremen, Hamburg, Hesse, North Rhine-Westphalia, Rhineland-Palatinate, Saarland, Schleswig-Holstein).

In sensitivity analyses, we evaluated the effect of vaccination before pregnancy by additionally including women who had their pregnancy first documented in the fourth quarter of the year and had been vaccinated during the adjacent third quarter. Additionally, in order to rule out that the comparison in VC across seasons was biased due to differences in included ASHIPs in analyses of the first three seasons (2014/15, 2015/16, 2016/17) compared to the last three seasons (2017/18, 2018/19, 2019/20), we reran the analysis solely including those 13 ASHIPs with available data in all six seasons.

## 3. Results

Table 1 shows the number of pregnant women identified as eligible for influenza vaccination during the influenza seasons under study and their distribution according to region and age. The considerably lower number of pregnant women in the early compared to the later seasons can be attributed to the fact that not all ASHIPs had provided the necessary data for inclusion into study for the earlier seasons. Consistently across seasons, the highest proportion of pregnancies was accounted for by age group 30 to <35 years (35 to 37%), followed by age groups 25 to <30 years (27 to 30%) and 35 to <40 years (18 to 20%). In addition, the proportion of pregnancies among women aged 30 years and above increased over the observation period (56.2% vs. 61.8% in 2019/20).

**Figure 1:**
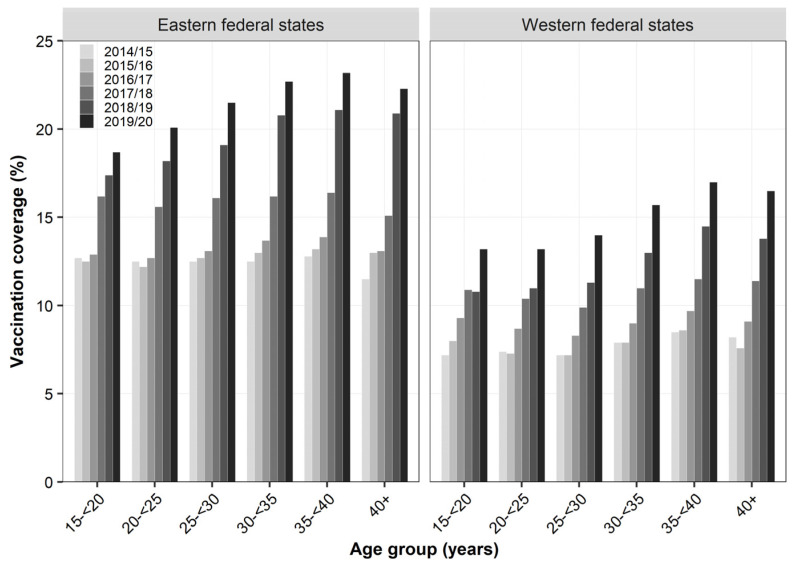
Influenza vaccination coverage (%) during pregnancy across region and age group based on outpatient claims data, seasons 2014/15 to 2019/20.

Overall, 16.6% of women who were pregnant during influenza season 2019/20 were vaccinated during that season (Table 2). Compared to season 2014/15, VC increased by 84%, while most of the increase occurred from season 2016/17 (VC: 9.9%) onwards (+68%). Women in eastern federal states were 40 to 60% more likely to have been vaccinated compared to women residing in western federal states. At the level of ASHIPs, variation in VC was 3.5-fold in season 2014/15 and continuously decreased to 2.2-fold in season 2019/20, with coverage ranging from 5.5% to 12.9% in 2014/15 and 13.3% to 29.5% in 2019/20 (data not shown). Consistently across seasons, coverage was highest among women aged 35 to <40 years (2019/20: 18.2%). Compared to 2014/15, VC particularly increased in age groups ≥30 years (+90% to +98%) compared to younger age groups (+64% to +75%). Overall, the higher VC in eastern compared to western federal states found in the total study population was similarly observed across all age groups (Figure 1). On a relative scale, the difference between the two regions slightly decreased among all age groups during the study period. However, women in eastern federal states were still 35% (40+ years) to 54% (25 to <30 years) more likely to have been vaccinated in season 2019/20 compared to their western counterparts. Despite this regional difference in coverage, the overall pattern across age groups was comparable between both regions with the highest coverage observed among women aged 35 to <40 years (23.2% and 17.0%, respectively).

Results remained virtually unchanged when we accounted for vaccination before pregnancy. Specifically, VC increased in all seasons by roughly 0.2 percentage points when we included women who were vaccinated in the third quarter and for whom prenatal care was first documented in the fourth quarter. Vaccination coverage remained unchanged when we restricted the study population to those 13 ASHIPs with available data in all six seasons under study. Specifically, vaccination coverage amounted to 9.0% (2015/16), 9.7% (2016/17), 11.6% (2017/18), 14.0% (2018/19) and 16.4% (2019/20).

## 4. Discussion

As an initial step towards the annual reporting of influenza VC among pregnant women in Germany, the present study provides data for seasons 2014/15 to 2019/20. Although we observed a continuous and marked increase in VC from season 2016/17 to 2019/20, coverage still remained low and, additionally, displayed considerable regional variation. In 2019/20, nine years after the endorsement of the official recommendation in 2010, more than 80% of pregnant women were unprotected and thus more vulnerable to influenza infection and possible severe complications.

In a previous claims-based study, Bätzing et al. reported VC for the years 2010 to 2014 [19]. In that study, 9.4% of women who were pregnant in 2014 were vaccinated against influenza, an observation that is perfectly in line with our findings among women who were pregnant during season 2014/15. Findings from earlier population-based surveys in Germany generally indicated a higher coverage, ranging from 11 to 20% during influenza seasons 2012/13 and 2013/14 [16,17,18]. In brief, among 1025 women, 23% reported vaccination against influenza during the 2012/13 season, with 16% receiving the vaccination while being pregnant [18]. Comparable results were obtained based on documented information in vaccination certificates from a cross-sectional study comprising 253 pregnant women from two perinatal centers, in which 19.5% had a recorded influenza vaccination during pregnancy [16]. In a nationwide web-based prospective cohort study conducted between 2012 and 2014, 11% of 838 women reported influenza vaccination during pregnancy [17]. Considering that these surveys were of a small sample size and, inherently due to their design, may be limited by selection and/or recall bias, we would expect a higher VC in these primary non-representative studies compared to the present large-scale secondary data analysis.

Strikingly, after years of relatively stable VC levels of ~9% (see also Bätzing et al., [19] for the years 2010 to 2014), coverage increased constantly from season 2017/18 onwards. Although it currently remains unclear whether this trend is of a sustained nature or merely represents a pronounced fluctuation, it is tempting to speculate that intensified information campaigns, as have been initiated and orchestrated by the Professional Association of Gynecologists in season 2017/18, have begun to bear fruit [21]. While the average VC was 9.3% up to season 2016/17 (2014/15 to 2016/17), it was 14.2% from season 2017/18 onwards (2017/18 to 2019/20). In brief, this campaign aimed to increase public awareness and knowledge on influenza illness and vaccination during pregnancy, e.g., through means of several press releases distributed over the whole influenza season, the publication of interviews as well as the expansion and improvement of information on the central information website of the Professional Association of Gynecologists. The increase in media response observed during and after this campaign may have contributed to the observed increase in VC. Secondly, VC may be partly attributed to the increase in age at pregnancy observed during the study period. Specifically, the shift in age distribution towards a higher proportion of pregnant women aged ≥ 30 years in conjunction with the generally higher VC in this age group may in part explain the overall increase in VC.

When comparing VC among pregnant women between industrialized countries, low to moderate coverage and high variations are observed. Based on data from nine EU/EEA Member States, the median VC was 25% in season 2016/17 and varied between 0.5% in Slovenia and 59% in Northern Ireland [2]. In the US, coverage estimated from annually conducted Internet panel surveys ranged between 40 and 50% between 2010/11 and 2017/18, before increasing to 61% in season 2019/20 [8,9]. The substantial between-country differences in VC may be partially attributed to different study designs to assess coverage, differences in communication activities supporting the recommendation, in vaccination systems, funding schemes, and different attitudes towards vaccination during pregnancy as well as the time evolved since endorsement of the recommendation, which may affect the acceptance of the vaccination and ultimately the coverage [18]. While in the US, influenza vaccination was recommended for pregnant women in the second trimester of pregnancy from 1997 and regardless of the trimester from 2014 onwards [22,23], it is recommended in Germany for a much shorter time. Interestingly, VC was equally low in the US within the first 10 years after endorsement of the recommendation (~15%; [24]) and thus comparable in size to the coverage currently observed in Germany.

Several factors have been shown to be associated with VC among pregnant women, including poor vaccine-related knowledge, vaccine safety concerns, mistrust in the vaccines, and perceptions related to the severity of disease [18,25,26,27,28,29]. Evidence further consistently emphasizes the crucial role of physicians, i.e., gynecologists, in increasing VC among pregnant women. Specifically, gynecologists are considered the most frequently used source to obtain information on influenza vaccination during pregnancy and studies have shown that women receiving a vaccination advice from a physician are more likely to be vaccinated [16,17,18,26,29].

In 2003, the World Health Assembly urged the Member States to increase influenza VC of all people at high risk, including the elderly and individuals with underlying diseases, and to attain VC among the elderly of at least 75% by 2010 [30]. In the EU/EAA region, countries have not managed to achieve the coverage target in any target group and the uptake of vaccination varies greatly between countries [2]. For Germany, two recent analyses similarly indicate that further efforts are needed to increase coverage of influenza vaccination in these target groups. Despite VC increasing among individuals aged ≥ 60 years the second season in succession, it reached on average 39% in season 2019/20 [15]. Among individuals with a chronic condition, VC ranged from 19% in individuals with multiple sclerosis to 44% in individuals with chronic kidney disease in season 2017/18 with a tendency towards a decline during the observation period [31].

Routinely collected health claims data offer the unique opportunity to estimate the uptake of vaccinations under real-world conditions based on large sample sizes. The present data is based on data from nearly all SHI-insured individuals, reflecting up to 80% of the total German population. Hence, selection bias is negligible compared to population-based surveys and studies based on single SHI funds. However, results have to be interpreted against the backdrop of the following limitations. First, the identification of pregnant women was based on the presence of two consecutive quarters with pregnancy-related health care services and, thus, based on continuous care through an SHI-authorized physician in at least two consecutive quarters of pregnancy. Pregnant women with a low compliance might therefore be underrepresented in the present study, which might have resulted in an overestimation of VC. Second, the majority of pregnant women usually continue their professional activities during the first and second trimester of pregnancy, which is the time most relevant for influenza vaccination. In Germany, some public employers and companies offer free on-site influenza vaccinations for their employees, which are administered through company medical officers and, therefore, are not included in the present data. Third, due to differences in the pseudonymization procedure between ASHIPs, it is currently not possible to follow-up individuals who move from one ASHIP region to another. Hence, we could not include women receiving two consecutive quarters of outpatient services for prenatal care in more than one ASHIP (e.g., due to moving between ASHIPs). However, it is unlikely that this could have exerted a noticeable effect on our results.

## 5. Conclusions

Although a constant increase in influenza vaccination uptake among pregnant women in Germany was noted from 2016/17 onward, vaccine uptake remains very low. Starting with this analysis, influenza VC among pregnant women in Germany will be monitored and reported on a yearly basis to evaluate the progress in achieving a higher coverage. Further efforts, i.e., involving intensified information through gynecologists and an improved understanding of barriers to vaccination through questionnaire-based studies, may guide the development and refinement of tailored strategies to support a sustainably improved coverage and reduce influenza and influenza-related complications during pregnancy.

## Figures and Tables

**Table 1:** Number and distribution of pregnant women eligible for influenza vaccination based on outpatient claims data, by region and age group, seasons 2014/15 to 2019/20.

	2014/15		2015/16		2016/17		2017/18		2018/19		2019/20	
	N	%	N	%	N	%	N	%	N	%	N	%
**Total**	481,197	100	509,897	100	600,660	100	626,135	100	615,331	100	614,239	100
**By region**												
Eastern federal states	124,286	25.8	128,706	25.2	131,938	22.0	129,251	20.6	124,873	20.3	121,216	19.7
Western federal states	356,911	74.2	381,191	74.8	468,722	78.0	496,884	79.4	490,458	79.7	493,023	80.3
**By age group**												
15 to <20	10,102	2.1	10,523	2.1	12,763	2.1	12,027	1.9	10,884	1.8	10,129	1.6
20 to <25	54,259	11.3	53,620	10.5	64,227	10.7	64,688	10.3	61,317	10.0	59,062	9.6
25 to <30	146,087	30.4	154,925	30.4	180,289	30.0	181,336	29.0	171,544	27.9	165,973	27.0
30 to <35	168,996	35.1	178,837	35.1	209,734	34.9	222,692	35.6	224,379	36.5	229,442	37.4
35 to <40	84,832	17.6	93,921	18.4	112,272	18.7	121,233	19.4	122,219	19.9	123,367	20.1
40+	16,921	3.5	18,071	3.5	21,375	3.6	24,159	3.9	24,988	4.1	26,266	4.3

**Table 2:** Influenza vaccination coverage (%) during pregnancy based on outpatient claims data, by region and age group, seasons 2014/15 to 2019/20.

	2014/15	2015/16	2016/17	2017/18	2018/19	2019/20
**Total (nationwide, all age groups)**	9.0	9.0	9.9	11.8	14.1	16.6
**By region**						
Eastern federal states	12.5	12.8	13.4	16.1	20.0	22.1
Western federal states	7.7	7.8	8.9	10.7	12.6	15.3
**By age group**						
15 to <20	9.0	9.4	10.3	12.3	12.6	14.8
20 to <25	8.8	8.5	9.5	11.4	12.4	14.7
25 to <30	8.8	8.7	9.4	11.2	12.8	15.4
30 to <35	9.0	9.2	10.0	12.1	14.6	17.1
35 to <40	9.4	9.7	10.6	12.5	15.8	18.2
40+	8.9	8.8	9.9	12.1	15.1	17.7

## Data Availability

Restrictions apply to the availability of the data. Data were obtained from the Associations of Statutory Health Insurance Physicians and are not publicly available.
